# Aetiology and Management of Acute Septic Arthritis and Prosthetic Joint Infection Presentations to Orthopaedics: An Evaluation of Tertiary Centre Performance

**DOI:** 10.7759/cureus.108830

**Published:** 2026-05-14

**Authors:** Calvin R Finlayson, Neta M Hansen, Sophie Stewart, Matthew Smith

**Affiliations:** 1 Trauma and Orthopaedics, Aberdeen Royal Infirmary, Aberdeen, GBR

**Keywords:** clinical audit, musculoskeletal infection, orthopaedics & traumatology, orthopedic implant-related infection, postoperative joint infection, prosthetic joint, septic arthritis, staphylococcus aureus

## Abstract

Background

Septic arthritis (SA) and prosthetic joint infection (PJI) are common emergency referrals to orthopaedics in the United Kingdom. These infections carry a significant risk of harm due to chondrolysis and arthropathy and are a potential source of sepsis, requiring prompt assessment, investigation, and treatment. A joint aspirate is routinely sent for microscopy, culture , and sensitivities to aid treatment. In suspected PJI, this must be taken under sterile conditions to reduce the risk of iatrogenic PJI. Empirical antibiotics should then commence per the British National Formulary (BNF) or local policy. Due to the risks of delayed or ineffective treatment, evaluating local practice may identify areas to optimise treatment delivery and efficacy.

Aims

This study will evaluate the incidence of SA and PJI within a tertiary orthopaedic centre over 12 months. The culprit organisms and sensitivities, delivery of timely and appropriate antibiotics, serum infection markers, length of stay (LOS), and mortality will be reviewed.

Methods

Admissions to the orthopaedic unit between November 2023 and October 2024 were screened using the ICD-10 codes for pyogenic arthritis, prosthesis infection, or infection following a procedure. Further review confirmed cases of SA or PJI, excluding unsuitable admissions. Each patient's electronic record was examined for demographics, serum infection markers, LOS, 30-day mortality, relevant imaging, time to aspirate relative to admission, culture results, and timing and type of antibiotics initiated. Antibiotics used were compared to sensitivities and BNF recommendations to determine efficacy.

Results

A total of 27 admissions with SA and 24 of PJI were seen, accounting for 51 (1.9%) of 2,670 total admissions, relative to a mean annual arthroplasty incidence of 1,204 across the health board. The median age of PJI patients was 72 years, significantly higher than the median SA patient age of 52 years (p<0.01). Median LOS was also higher in PJI patients at 13.5 days compared to nine days in SA (p=0.036). Most infections were hip and knee joints at nine (33.3%) cases in SA, and 11 (45.8%) total knee replacements in PJI cohorts. *Staphylococcus aureus* was the most prevalent organism in each group; however, 11 (40.7%) of the SA aspirate cultures were negative. The median aspiration time, relative to admission, was 5.6 and 10.2 hours in SA and PJI, respectively. The median time to initiate antibiotics was 7.1 and 17.9 hours in SA and PJI, respectively. The majority of admissions received appropriate antibiotics at 20 (74.1%) of SA and 17 (70.8%) of PJI patients. Culture results confirmed sensitivity to recommended antibiotics in 14 (51.8%) of SA and 18 (75%) of PJI cases. The most initiated antibiotic overall was flucloxacillin.  There was no significant difference in infection markers.

Conclusion

This study finds that PJI patients were typically older, with a greater LOS and comorbidity. Large joint infections, such as hips and knees, were the commonest presentation. The majority of each cohort was appropriately treated per the BNF, with *S. aureus* being the commonest cause. Factors such as prehospital antibiotics may reduce the bacterial yield of aspirations. Both groups show prolonged time taken to aspirate and treat, in which quality improvement interventions may reduce. Other centres may benefit from similar evaluations of the infective microbiome and the speed and efficacy of septic joint treatment.

## Introduction

Joint infections are common emergency referrals to orthopaedic units and rheumatology departments from both primary care and emergency departments for rapid assessment, investigations, and treatment. If not correctly diagnosed and treated, septic arthritis (SA) can lead to chondrolysis, arthropathy, sepsis, and ultimately death as a consequence of septic shock. Common differential diagnoses for the presentation of an acutely pyrexic patient with a painful and swollen joint can include gout, pseudogout, cellulitis, and hemarthrosis. Performing a joint aspiration for investigations such as culture and microscopy for bacterial identification is key to identifying SA, rather than mimicking pathology. In those patients with prosthetic joints, such as hip, knee, and shoulder arthroplasties, aspirating the joint to provide this information carries a significant risk of introducing infection into the potentially otherwise aseptic synovium, potentially leading to new prosthetic joint infection (PJI) and the requirement to undergo a revision, removal of metalwork, or disarticulation. In such cases, the British Orthopaedic Association recommends sterile aspiration in theatre to reduce the risk of introducing iatrogenic infection [1].

Treatment for suspected SA or PJI involves the prompt administration of antibiotics such as flucloxacillin or clindamycin, per the British National Formulary [2], after aspiration for a minimum of four weeks, as guided by local infectious diseases or microbiology teams. Patients who present with septic shock or are acutely deteriorating should have antibiotics administered promptly without any undue delay due to the logistical arrangement of aspiration. Given the significant impact on patients of the above complications, investigation into how such patients are managed may identify avenues for improvement and determine if the current recommendations remain effective in the microbiome and setting of a British major trauma centre.

This study sets out to evaluate the incidence of native joint SA and PJI admissions to the orthopaedic department of an acute tertiary unit over 12 months. Their admission blood tests, culprit organisms and sensitivities, and delivery of appropriate antibiotics were assessed to determine if current practices provide effective and timely treatment. To assess the impact of these emergency patients on a trauma unit, the corresponding length of stay and mortality associated with SA and PJI will also be examined.

## Materials and methods

**Figure 1:**
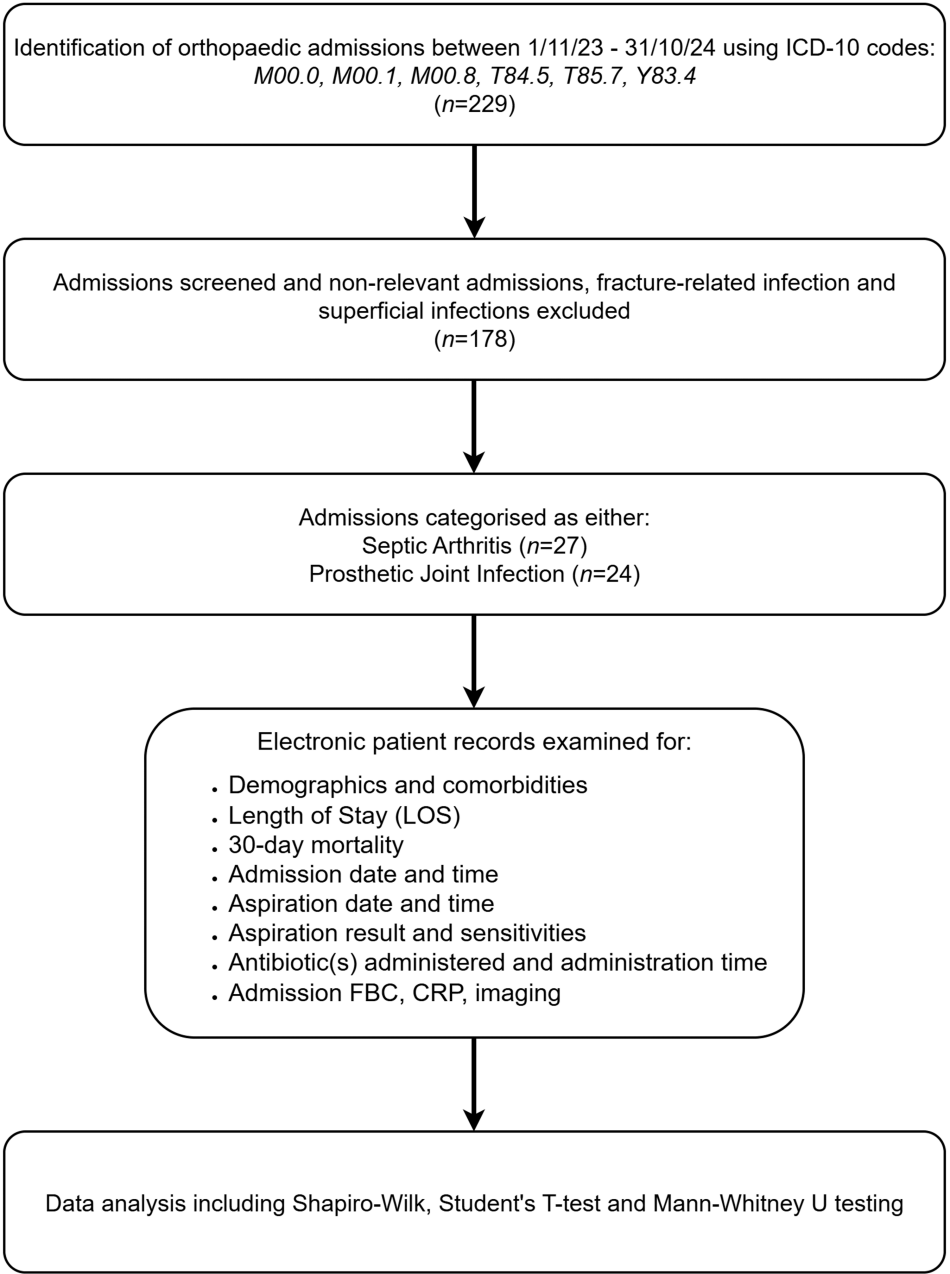
Data collection and handling process FBC: Full blood count, CRP: C-reactive protein, ICD-10: International Classification of Diseases, 10th Revision

Utilising the International Classification of Diseases, 10th Revision (ICD-10) diagnostic codes, admissions to orthopaedic wards at Aberdeen Royal Infirmary between November 2023 and October 2024 were screened for pyogenic arthritis, prosthesis infection, or infection following a procedure. Further screening identified cases of native-joint SA and PJI and excluded any falsely positive coding. Superficial infections or fracture-related infections were excluded. Of these, each patient's electronic patient record (EPR) was examined to determine age and sex, comorbidities, length of stay (LOS), mortality within 30 days of admission, time to sampling, aspirate culture result, antibiotic administration time, and type of antibiotic administered. Additionally, their full blood count (FBC), C-reactive protein (CRP), and any relevant imaging were examined for evidence of infection. The causative organisms and corresponding sensitivities and resistances of admissions with positive joint aspiration cultures were analysed to determine the effectiveness of the initiated antibiotics. The process of identifying organisms and their susceptibilities in the microbiology laboratory was carried out via matrix-assisted desorption/ionisation-time of flight mass spectrometry (MALDI-TOF MS), followed by susceptibility analysis using the VITEK® 2 system (bioMérieux S.A.; Auvergne‑Rhône‑Alpes, France) [3]. The data collection process is described in Figure 1. The total number of admissions to the unit during the analysis period was established by enquiry with the Grampian Health Intelligence Team.

The time of admission was determined as the time electronically admitted to the unit via any referral pathway. The time of aspiration was determined by the collection time recorded in the laboratory record. The earliest time of antibiotic administration was determined either as recorded via a scanned paper prescription available on the EPR from emergency care, or the earliest administration recorded on the Hospital's Electronic Prescribing and Medicines Administration (HEPMA). Only the initial aspiration and initial antibiotics were recorded. Duration of treatment was confirmed either via HEPMA or on the discharge document from each admission.

The antibiotic initiated per admission was compared to both the BNF recommendations and any corresponding positive culture per admission. An appropriate antibiotic was determined as any of the BNF recommendations for any subtype of SA or PJI: flucloxacillin, clindamycin, vancomycin, teicoplanin, cefotaxime, or ceftriaxone - depending on the patient's allergy status or suspected organisms on assessment. Radiological investigations, such as X-rays, were reviewed for qualitative evidence of infection, as reported by a radiologist or reporting radiographer.

Once collected, each dataset per infection cohort underwent Shapiro-Wilk testing to determine their distribution, before undergoing two-tailed Mann-Whitney U or Student's t-test testing to test for significant differences in each domain as appropriate. All data handling and processing was anonymised and completed in Microsoft 365 Excel (Microsoft® Corp., Redmond, WA).

To evaluate annual arthroplasty incidence within the local health board, an enquiry was made to the Scottish Arthroplasty Project [4] to determine the incidence of primary and revision arthroplasty for any joint in NHS Grampian.

## Results

A total of 27 (1%) admissions for SA and 24 (0.9%) for PJI were observed between November 2023 and October 2024, producing similar incidences of joint infection-related admissions per year. The total number of admissions for any cause to the trauma unit over the 12 months examined was 2,670.

The median age between SA patients and PJI patients was significantly different at 52 and 72, respectively (p<0.001). The median LOS was also significantly different, with nine days in cases of SA, and 13.5 days for cases of PJI (p=0.037). The proportion of male sex patients between each arm was comparable at 15 (60%) of the SA and 13 (56.5%) of the PJI cohorts. The only 30-day mortalities recorded in this study were two patients with infected joint prostheses. The median number of comorbidities per patient was found to be significantly higher in PJI patients at four comorbidities compared to two (p=0.04). A breakdown of the patient characteristics is seen in Table 1.

**Table 1:** Patient characteristics

Patient Characteristics	Septic Arthritis		Prosthetic Joint Infection		Total	
	n	%	n	%	n	%
Sex						
Male	15	60	13	56.5	28	58.3
Female	10	40	10	43.5	20	41.7
Comorbidities						
Hypertension	7	28	9	39.1	16	33.3
Total Knee Replacement	2	8	8	34.8	10	20.8
Total Hip Replacement	2	8	8	34.8	10	20.8
Malignancy (Any)	3	12	4	17.4	7	14.6
Intravenous Drug Use	4	16	2	8.7	6	12.5
Atrial Fibrillation	2	8	2	8.7	4	8.3
Type Two Diabetes Mellitus	2	8	2	8.7	4	8.3
Gastroesophageal Reflux Disease	1	4	3	13	4	8.3
Anxiety	2	8	2	8.7	4	8.3
Septic Arthritis	3	12	0	0	3	6.3
Heart Failure	0	0	3	13	3	6.3
Obesity	0	0	3	13	3	6.3
Total Shoulder Replacement	1	4	2	8.7	3	6.3
Chronic Obstructive Pulmonary Disorder	1	4	2	8.7	3	6.3
Osteoarthritis	1	4	2	8.7	3	6.3
Deep Vein Thrombosis	2	8	1	4.3	3	6.3
Chronic Kidney Disease	1	4	2	8.7	3	6.3
Musculoskeletal Injury	2	8	0	0	2	4.2
Pulmonary Embolism	2	8	0	0	2	4.2
Transient Ischaemic Attack	2	8	0	0	2	4.2
Gout	0	0	2	8.7	2	4.2
Depression	0	0	2	8.7	2	4.2
Benign Prostatic Hypertrophy	0	0	2	8.7	2	4.2
Prosthetic Joint Infection	1	4	0	0	1	2.1
Nil	2	8	0	0	2	4.2
Not Recorded	2	8	1	4	3	6.3
n = 2	8	32	4	17	12	25
n = 3	6	24	2	9	8	16.7
n = 4	2	8	4	17	6	12.5
n > 4	3	12	9	39	12	25

The most frequent presentation in SA cases was both infected native hip and knee joints at nine (33.3%) cases each (Table 2), where in PJI cases, infected total knee replacements were the majority at 11 (45.8%) cases (Table 3).

**Table 2:** Presentations of septic arthritis (n=27)

Site of Infection	Count: n (%)
Hip	9 (33.3%)
Knee	9 (33.3%)
Wrist	4 (14.8%)
Elbow	2 (7.4%)
Other	2 (7.4%)
Shoulder	1 (3.7%)

**Table 3:** Presentations of prosthetic joint infection (n=24)

Site of Infection	Count: n (%)
Total Knee Replacement	11 (45.8%)
Total Hip Replacement	8 (33.3%)
Hip Hemiarthroplasty	4 (16.6%)
Reverse Total Shoulder Replacement	1 (4.2%)

**Figure 2:**
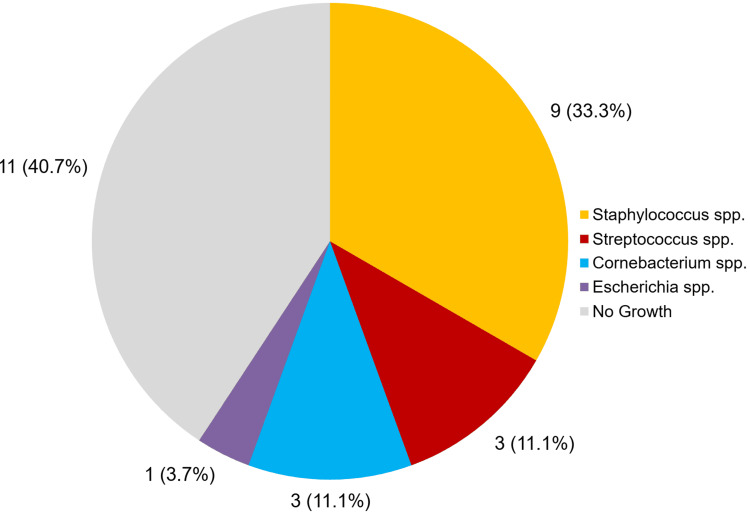
Culture results from septic arthritis aspirates (n=27)

**Figure 3:**
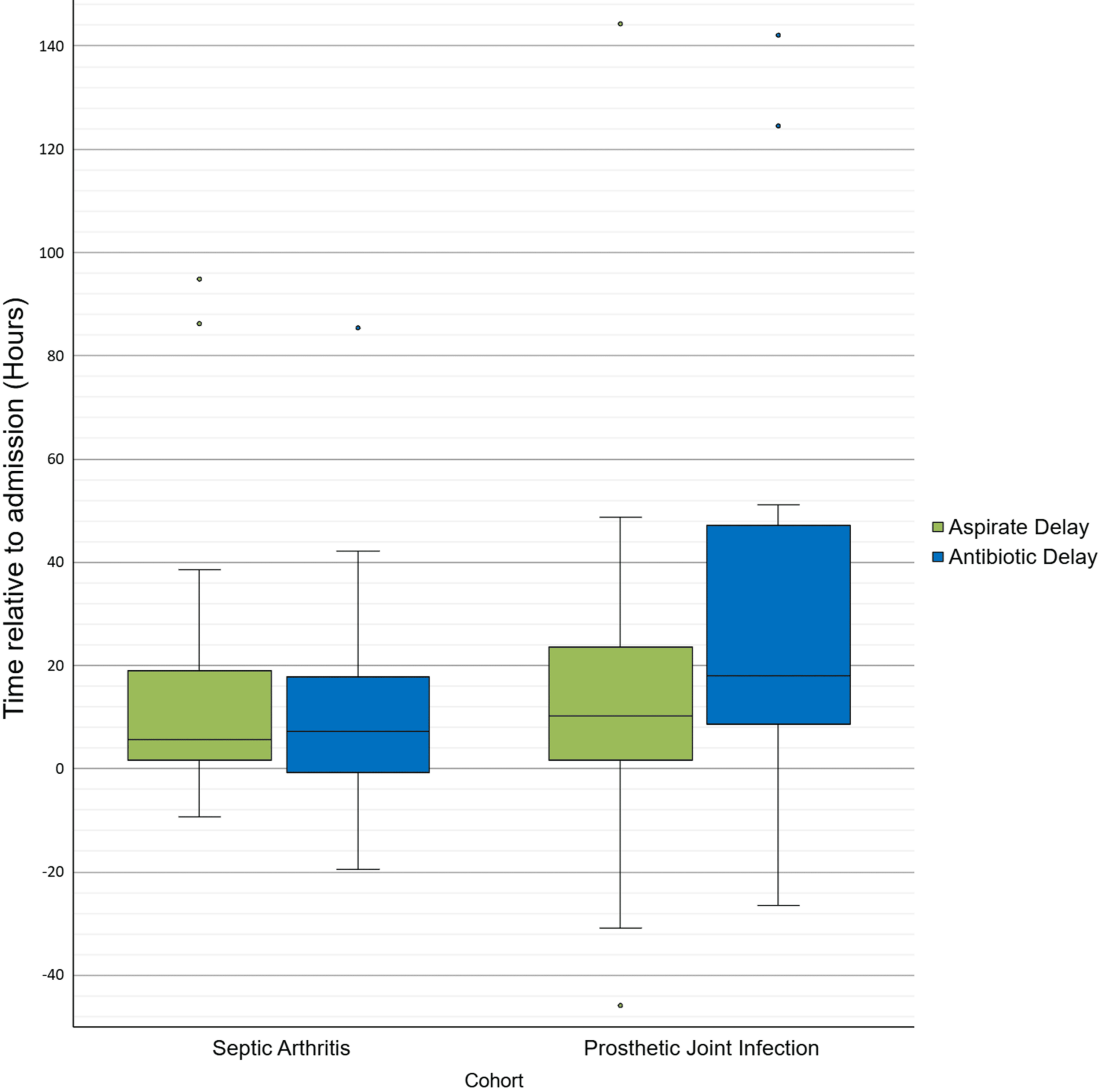
Time to aspiration and initial antibiotic administration per cohort

Of those diagnosed with SA, aspirate culture results yielded no growth in 11 (40.7%) cultures, but the most prevalent species of bacteria isolated was *Staphylococcus *spp. in nine (33.3%) culture results (Figure 2). The median time, relative to admission, for these patients to undergo aspiration was 5.6 hours and 7.1 hours to receive antibiotics (Figure 3). The most commonly initiated antibiotic in this group was flucloxacillin in 18 (66.7%) admissions (Table 4). 

**Table 4:** Initial antibiotic therapy used in septic arthritis and prosthetic joint infection admissions

Antibiotic Initiated	Count: n (% of Cohort)	
	Septic Arthritis Patients (n=27)	Prosthetic Joint Infection Patients (n=24)
Flucloxacillin	18 (66.7%)	12 (50%)
Benzylpenicillin	6 (22.2%)	1 (4.2%)
Co-amoxiclav	3 (11.1%)	0
Vancomycin	2 (7.4%)	1 (4.2%)
Amoxicillin	2 (7.4%)	3 (12.5%)
Daptomycin	2 (7.4%)	0
Metronidazole	1 (3.7%)	1 (4.2%)
Teicoplanin	0	2 (8.3%)
Gentamicin	1 (3.7%)	0
Cotrimoxazole	0	1 (4.2%)
Cefuroxime	0	1 (4.2%)
Ceftriaxone	0	1 (4.2%)
Daptomycin	0	1 (4.2%)

**Figure 4:**
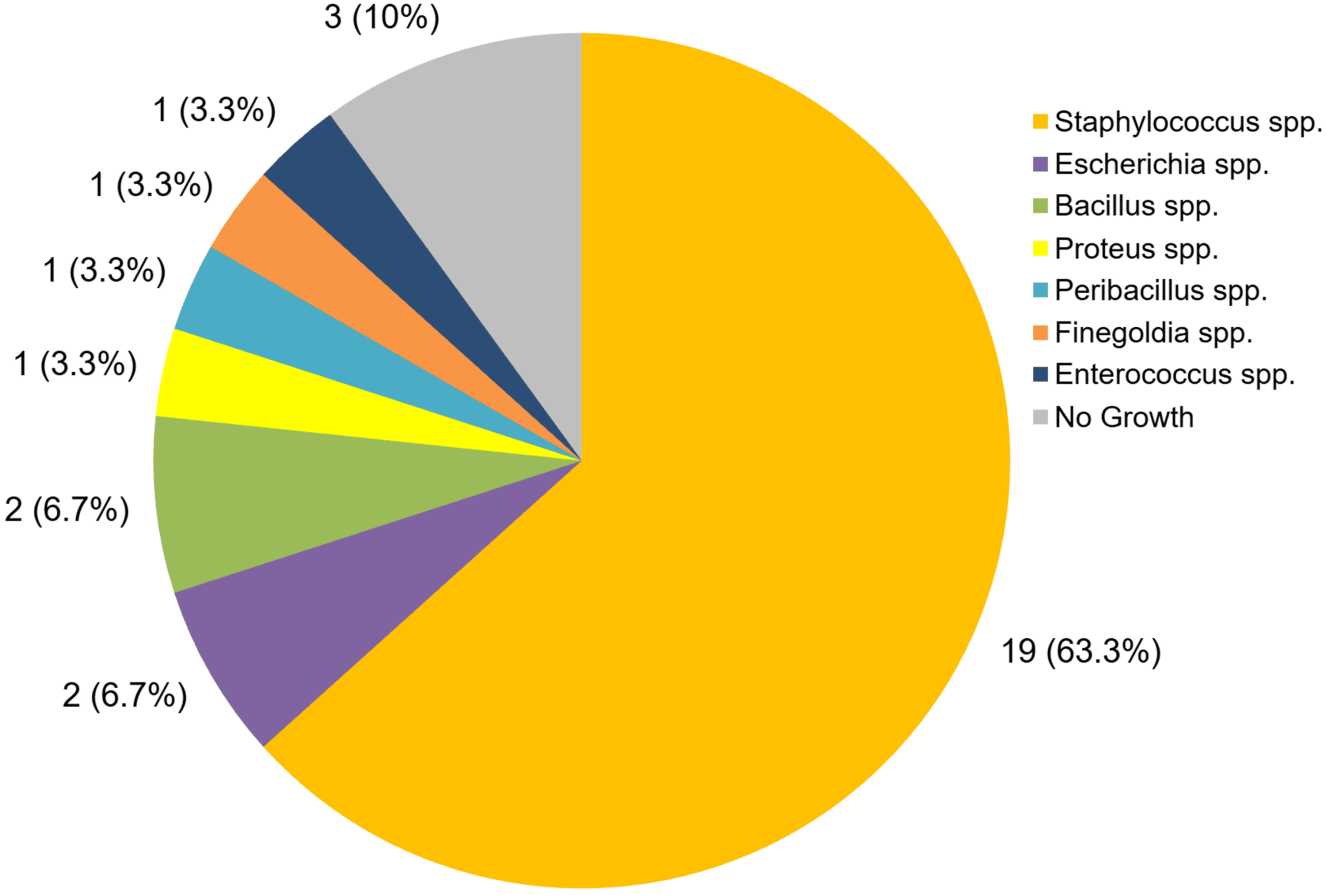
Culture results from prosthetic joint infection aspirates (n=30)

In cases of PJI, aspirations performed produced proportionally more positive culture results than SA, with 27 (90%) compared to 16 (59.2%) positive results in SA. A majority of 19 (64%) of aspirate culture results isolated a *Staphylococcus *spp. (Figure 4). The median times of aspiration and initiating antibiotics, relative to admission, in PJI patients were 10.2 and 17.9 hours, respectively (Figure 3). There was no statistically significant difference seen in the time taken to aspirate the relevant joint between SA and PJI (p=0.064), but significance was seen in the delay to start antibiotics (p=0.01). Fifty percent of PJI patients' initial treatment included flucloxacillin (Table 4). 

In SA patients, the number of admissions commencing any appropriate antibiotic per the BNF was 20 (74.1%). By comparison, PJI admissions had appropriate antibiotics initiated in 17 (70.8%) of cases. A breakdown of antibiotics as initiated is detailed in Table 4. Where the recommended antibiotics were administered, these were confirmed to be sensitive in 10 (37%) and 13 (54.2%) of the positive SA and PJI cultures, respectively, and the majority of each cohort received a minimum of four weeks of antibiotics (Table 5).

**Table 5:** Adherence with the British National Formulary (BNF) recommendations

Recommendation Adherence	Septic Arthritis (n=27)	Prosthetic Joint Infection (n=24)
Admissions with a positive aspirate culture	13 (48.1%)	20 (83.3%)
Admissions initiating BNF recommended antibiotic	20 (74.1%)	17 (70.8%)
Cultures sensitive to BNF recommendation	14 (51.8%)	18 (75%)
Admissions receiving recommended antibiotics with aspirate culture-proven sensitivity	10 (37%)	13 (54.2%)
Admissions receiving at least 28 days of antibiotics	19 (70.4%)	20 (83.3%)

In establishing significance, Shapiro-Wilk testing was first performed on all datasets to determine normality and direct further analysis. The age of SA patients, admission haemoglobins, white cell counts, and neutrophil counts were found to be normally distributed, while LOS, comorbidity count, aspiration time, and antibiotic administration time were found to be non-parametrically distributed in both SA and PJI patients. The age, CRP, and comorbidity count of PJI patients were all found to be non-parametrically distributed, as p<0.05, and the null hypothesis that the data are normally distributed is rejected. Correspondingly, the datasets then underwent Mann-Whitney U testing to establish statistical significance between the observed median values in the non-parametric distributions seen. In this analysis, a statistical difference where p<0.05 was observed in the difference of medians in age, LOS, comorbidity count, and antibiotic administration time between SA and PJI cases. The median time from admission to aspiration was not found to be statistically significantly different between the SA and PJI groups (p>0.05). Full statistical analysis is shown in Table 6.

**Table 6:** Statistical analysis of results including significance LOS: Length of stay; PJI: Prosthetic joint infection; SA: Septic arthritis

Data	Cohort	Median	Standard Deviation	Minimum	Maximum	Range	Shapiro-Wilk Result (p)	Mann-Whitney U Result (p)
Age (Years)	SA	52	16.5	27	85	58	0.295	<0.01
	PJI	72	11.6	37	84	47	<0.01	
LOS (Days)	SA	9	20.0	1	96	95	<0.01	0.036
	PJI	13.5	20.4	1	83	82	<0.01	
Aspirate Delay Relative to Admission (Hours)	SA	5.6	25.7	-9.3	94.9	104.2	<0.01	0.638
	PJI	10.2	35.2	-45.9	144.3	190.2	<0.01	
Antibiotic Delay Relative to Admission (Hours)	SA	7.1	20.4	-19.5	85.4	104.9	<0.01	0.010
	PJI	17.9	38.4	-26.5	142.1	168.6	<0.01	

Radiological imaging was performed in the majority of each cohort, but evidence of infection was only reported in a minority of cases. X-rays were performed in 22 (81.4%) admissions with SA, compared to 17 (70.8%) of the PJI admissions. Of these X-rays, radiological evidence of infection was only described in four (23.5%) of the SA and four (14.8%) of the PJI images. Routine biochemical assessment of infection markers was carried out on all patients and is shown in Table 7. This demonstrated elevated average results for white cell count, neutrophil count, and CRP compared to local reference values; however, there was no significant difference between infection markers of the groups.

**Table 7:** Biochemical results on admission WCC: White cell count, CRP: C-reactive protein, SD: Standard deviation, SW: Shapiro-Wilk

Investigation	Reference Value	Septic Arthritis	SD	SW Test (p)	Prosthetic Joint Infection	SD	SW Test (p)	Significance (p)
Haemoglobin (g/L) (Mean)	108-156	120	20	0.935	123	22	0.903	0.582
Mean WCC (x10^9^/L)	4-10	11	4	0.903	11	5	0.192	0.949
Mean Neutrophil Count (x10^9^/L)	1.5-7	9	3	0.359	9	5	0.095	0.979
Median CRP (mg/L)	0-4	162	96	0.320	98	124	0.003	0.406

The majority of patients in each group underwent plain-film radiography on admission, as demonstrated in Table 8. When examined by a radiologist or reporting radiographer, the number of films reported with evidence of infection was much lower, with only four admission X-rays demonstrating this in both cases of SA and of PJI.

**Table 8:** Radiological evidence of infection SA: Septic arthritis; PJI: Prosthetic joint infection

Admission Investigation	SA (n)	SA (%)	PJI (n)	PJI (%)
X-rays performed	23	85.2	17	70.8
X-rays demonstrating joint infection	4	14.8	4	16.7

The annual incidence of arthroplasty and revision arthroplasty in NHS Grampian was derived from the data gathered by the Scottish Arthroplasty Project [4]. The mean annual number of primary arthroplasties performed was 1,204, while the mean number of revisions performed was 118 within the health board between 2023 and 2024. The breakdown of this data can be seen in Table 9. 

**Table 9:** Annual incidence of primary and revision joint arthroplasty in NHS Grampian Data courtesy of the Scottish Arthroplasty Project [4]. ARI: Aberdeen Royal Infirmary, DGH: Doctor Gray's Hospital, WGH: Woodend General Hospital

Year	2023			2024			Annual Mean
Hospital	ARI	DGH	WGH	ARI	DGH	WGH	
Primary Arthroplasty							
Hip	106	81	594	60	27	362	615
Knee	4	82	587	5	20	333	516
Shoulder	14	0	44	13	0	57	64
Other	2	0	4	5	0	7	9
Total	126	163	1229	83	47	759	1204
Revision Arthroplasty							
Hip	37	1	39	23	0	37	69
Knee	4	2	32	12	0	27	39
Shoulder	1	0	5	1	0	7	7
Other	0	0	3	0	0	5	4
Total	42	3	79	36	0	76	118

## Discussion

In this paper, we have highlighted the key distinctions in those patients with SA or PJI, in their management in this centre. Similar to national trends of multimorbidity in the elderly, both infections affect patients of increased age, which is associated with an increased incidence of comorbidities [5].

The pathogenic yield of joint aspirations also differed between the two groups, with SA cases having a lower positive culture rate at 48.1%, compared to a 83.3% positive culture growth rate for PJI aspirations. There are a number of factors thought to contribute to this, such as the use of prehospital antibiotics and sampling technique. While the British Society for Rheumatology (BSR) is undergoing a review of the 2006 recommendations of the combined working group of BSR & British Health Professionals in Rheumatology (BHPR), British Orthopaedic Association (BOA), Royal College of General Practitioners (RCGP), and British Society for Antimicrobial Chemotherapy (BSAC), this guideline explicitly states that the aspiration of the hot swollen joint to aid diagnosis is required and should be done before the administration of antibiotics unless significantly unwell [6]. By reducing antibiotic exposure before aspiration, a higher proportion of viable microorganisms will be available for culture within the laboratory setting [7]. In clinical practice, several patients present for acute assessment of a septic joint after already having received a course of oral antibiotics from primary care, which is suspected to contribute to the apparent lack of bacteria found on aspirates in native-joint SA patients. While it is noted that a negative culture does not exclude septic arthropathy in the context of other clinical findings, such as pain, joint pyrexia, the inability to mobilise the affected joint or systemic upset, it cannot be possible to exclude cases from this study, which may otherwise be a reactive, inflammatory or idiopathic arthropathy of another cause, which was not excluded by the treating team during the admission. A negative culture may also be the result of poor aspiration technique, as locally this is performed without ultrasound guidance to confirm interarticular needle placement rather than sampling a neighbouring bursa.

Our examination of infective biomarkers here, such as WCC and CRP in this study, has shown that an elevation is observed; however, this alone cannot be used to help distinguish a case of SA from PJI. Other investigations that may be useful to differentiate between these and other pathologies could include erythrocyte sedimentation rate (ESR) or crystal microscopy, where gout or pseudogout may be suspected; however, these were not examined in this study. An additional factor that predisposes prosthetic joints to infection is the presence of a biofilm adherent to the prosthesis, offering an area with poor antibiotic exposure and an extracellular matrix conferring protection from the host immune system that is favourable for growth, and this is a well-known phenomenon [8].

In performing the laboratory analysis of these cultures, it is important to highlight that different centres may use different methodologies, which may alter the lead time to receiving and then acting upon the results. In the Aberdeen Royal Infirmary, the cultured organisms are first identified through MALDI-TOF MS, where different organisms are identified by comparing the samples' proteomic mass spectra to reference values of different bacterial species. To then identify sensitivities and resistances, the VITEK® 2 system utilises several cassettes containing reagents and antimicrobials that produce coloured reactions sensitive to the metabolic processes involved in cell death. Once samples are loaded, the machine runs a carousel whereby each sample is regularly checked with an optical sensor to determine if and when these reactions occur. Therefore, positive reactions can indicate sensitivities to specific known antibiotics within the wells of a cartridge, in a process which can be as rapid as six to eight hours from testing the sample, according to the manufacturer [9]. This time may be a limiting factor in the ability to provide tailored antimicrobial therapy, but it should not affect the implementation of empirical antibiotics.

In the culture results from this study, *Staphylococcus aureus* was the most common organism grown in both patient groups, which is consistent with both historic and recent literature discussing the aetiology of these infections [10,11]. While *S. aureus* is widely implicated as a causative organism in SA and PJI, several other organisms, such as methicillin-resistant *S. aureus* (MSRA), *Streptococcus *spp., *Neisseria gonorrhoeae*, and, rarely, fungal agents, are known to cause joint infection via haematogenous spread, as described by He et al. [11]. Only bacterial agents were found on aspirate cultures during this investigation.

Antibiotic route and duration also remain a controversial area in the treatment of SA and PJI. The 2006 BSR & BHPR, BOA, RCGP, and BSAC guidelines recommend two weeks of intravenous (IV) antibiotics before switching to oral antibiotics for a further four weeks [6]. Local guidance from Right Decisions used by NHS Grampian suggests instead that patients should receive IV flucloxacillin in native joints with the addition of rifampicin in prosthetic joints, but defers the duration of treatment to local specialists [12]. In local practice, this discussion with the infectious diseases team is standard, and such an approach is recommended by the BOA as part of MDT management of such patients [1]. As part of such an approach, the Outpatient Antibiotic Therapy (OPAT) service plays a key role in reducing inpatient LOS and benefits patients by allowing them to receive treatment from local centres, increasing patient independence and reducing the associated costs of inpatient care. Mohammed et al. performed a systematic review and meta-analysis investigating the role of OPAT in reducing associated costs, compared to those receiving inpatient care [13]. They found an average saving of $5,436.73 (approximately £4,116.19) in the cost of parenteral antimicrobial treatment per episode of care.

This study has shown that both SA and PJI patients experienced significant delays in joint aspiration and antibiotic initiation. Notably, the time taken to commence antibiotics was significantly longer in the PJI patients, which may reflect the challenges in coordinating theatre-based aspirations and the availability of revision implants to complete single-stage revisions where aspiration and revision occur in the same theatre session. In contrast to these patients requiring theatre time and staffing, where native knee infections are suspected, these are performed under aseptic conditions in an assessment room or the emergency department by the reviewing on-call orthopaedic registrar, without ultrasound guidance, which is likely to reduce the median time to aspiration in native joints. While we did not demonstrate a significant difference in the timing of aspiration, this has a downstream effect of also delaying the administration of antibiotics until this has been performed in stable patients. The British Orthopaedic Association Standard for Trauma (BOAST) guideline on PJI recommends that those acutely unwell with PJI should undergo drainage, or other procedures such as debridement, within six hours of presentation [14]. In this evaluation, the clinical parameters of patients at presentation were not recorded, although this may be a useful metric in highlighting those in need of early drainage and debridement and formulating local service pathways to expedite such procedures in the acutely unwell. This study also did not assess service delivery metrics such as emergency department waiting times or ambulance arrival delay time prior to initial assessment, which may further add to the delays observed relative to admission to the orthopaedic ward.

In performing statistical analysis on the data obtained, it was important to ensure that the appropriate tests were utilised, and as such, each dataset was examined with Shapiro-Wilk testing to determine where non-parametric tests should be facilitated. In those characteristics, such as median LOS and median delay time, utilising the mean would not accurately describe the populations due to the presence of a minority of significant outliers; hence, Mann-Whitney U testing was appropriate for both those non-parametric data and where the median was subject to analysis in order to enhance statistical conclusions.

Full analysis in this study was impaired by a small number of documentation inconsistencies, such as missing prescribing data on HEPMA, as well as the inconsistent use of HEPMA by all departments involved in the care pathway of these patients. HEPMA was formally introduced to the centre in June 2023; however, implementation by individual wards took place over several months thereafter, coinciding with the start of the study period. While this prescribing tool has now undergone an effective roll-out, it was found that latterly patients had a more complete archive of previous drug administrations to reference compared to those in the initial weeks.

Overall, this study emphasises the need for streamlined protocols in such emergency presentations of SA or PJI. Performing such examinations of how a unit operates is useful both as surveillance of the local microbiome and in monitoring the effectiveness of the current empirical treatments. While, in this case, the orthopaedic unit has recently made steps to improve the sensitivity of their aspirations by providing general practitioner (GP) advice via discharge documents, pathways that highlight those at risk of deterioration or development of septic shock could improve recognition of the urgency to treat cases of SA and PJI.

In addition to prescribing data inconsistencies noted previously, there are a number of other limitations that may also influence the data. While the methodology employs ICD-10 coding, which is commonly available to most hospitals, it relies on manual data entry by medical administration coders based on information available on the discharge document. Any errors or unclear documentation regarding the diagnosis or presentation of the patient may lead to the exclusion of otherwise suitable cases for this study. This also introduces a latency period where, until such coding is completed, this methodology cannot be used to complete real-time case analysis, and an alternative prospective methodology may be able to circumvent this. Another limitation is that this study relies on the accurate timing of events in the patient record. It may be the case that patients are not admitted electronically to the orthopaedic ward but are physically present due to the workload of the hospital flow coordination team, leading to a lag or leap in their admission time and altering the time to aspiration and antibiotic administration relative to this pivotal data point. Such discrepancies in the specificity of times recorded for admission, aspiration, and antibiotic administration times are difficult to quantify in a retrospective study; however, both cohorts are susceptible to this.

While this study has been able to capture patients managed within the main orthopaedic unit, there is a known cohort of patients that it will not necessarily capture. Patients with low-grade PJI may also present to the elective orthopaedic service on follow-up of their procedures and undergo aspiration, single-stage revision, or debridement, antibiotics, and implant retention (DAIR) procedures in a separate elective unit. This would reflect a small minority of cases, as patients with a significant symptom burden or deterioration would be expected to be managed via emergency inpatient admissions; however, given the relatively small case numbers examined, additional cases may influence results, and subsequent examinations of both hospitals may reflect the true burden of SA and PJI in the district. Additionally, further data gathering from a greater time period would offer the chance to observe potential trends in joint infection management and strengthen the statistical significance of the derived results.

## Conclusions

This study identifies that those presenting with PJI were typically older and required a longer inpatient admission, typically 4.5 days longer in this context. Large joints were the most commonly seen infections in both native and prosthetic joints, such as hips and knees. There was some observed mortality within the PJI group; however, this was not statistically significant due to the small number observed. The cases of SA observed were less likely to have a positive aspirate to confirm an infection and diagnosis, but the majority of both the SA and PJI groups were treated per BNF guidance. The aspirate positivity rate of prosthetic joint admissions was much higher than that of SA at 20 (83.3%) admissions versus 13 (48.1%) admissions, respectively; however, each arm demonstrates that *Staphylococcus *spp. are the commonest culprit organism. Both arms demonstrated elevated serum infective markers, and low sensitivity of X-rays to infective changes in acute presentations of either infection.

Presentations of SA and PJI were both hindered by delayed aspiration and delayed delivery of the recommended empirical antibiotics. PJI suffered the greatest delay, with a median delay of 10.9 hours to undergo aspiration and 17.9 hours to receive antibiotics. The median aspiration of both groups occurs before their significantly different respective median antibiotic administration times. These observed delays may be reducible with local quality improvement initiatives, and other centres may benefit from conducting similar analysis of promptness and effectiveness of treatment.
